# Practical Points

**Published:** 1907-07-13

**Authors:** 


#### The Workmen's Compensation Act.

### PRACTICAL POINTS.

### EMPLOYERS' LIABILITY INSURANCE.

The clerk to a provincial hospital writes :—

My committee will be much obliged if you will inform me whether London hospitals are making any special features of their insurances other than in respect of the ordinary paid staff and of claims therefrom under :—

The Workmen's Compensation Acts, 1897, 1900, and 1906.
Employers' Liability Act, 1880.
Fatal Accidents Act, 1846.
Common Law, which, I presume, include all general liabilities.

My committee question whether they should not insure against all diseases and illnesses which can be attributed in any shape to accident which may happen to their staff in the course of their duties.

I assume it is necessary to insure probationers (who receive no salary, but are found with board and lodgings) and also the clerk.

---

In reply to our correspondent's inquiry, we believe it to be the fact that the only illness attributable to accident covered by the Act, for which hospital authorities would probably be liable, is blood poisoning incurred in the discharge of the duties of members of the staff. We believe that several hospitals have arranged with insurance companies to cover this risk in the ordinary rates which they are charging for insurance under the Acts mentioned. We believe it to be necessary to insure probationers, but we doubt if the clerk, who does not reside on the premises, and is a professional man engaged in his own office, would be a member of the staff to be insured under the Act.

